# “It is more Important than food sometimes”; Meanings and Functions of Music in the Lives of Autistic Adults Through a hermeneutic-phenomenological Lense

**DOI:** 10.1007/s10803-022-05799-2

**Published:** 2022-11-02

**Authors:** Kaja Korošec, Walter Osika, Eva Bojner-Horwitz

**Affiliations:** 1 Centre for Psychiatry Research, Department of Clinical Neuroscience, Karolinska Institute, Norra stationsgatan 69, plan 7, 113 64 Stockholm, Sweden; 2 Department of Music, Pedagogy and Society, Royal College of Music, Stockholm, Sweden; 3 Center for Social Sustainability, Institution of Neurobiology, Care Sciences and Society, Karolinska Institute, Stockholm, Sweden

**Keywords:** Autism, hermeneutic phenomenology, music, negative experiences, subjective perspectives, well-being

## Abstract

Subjective experiences of autistic adults remain under-researched, especially in the field of art. To learn more about their uses and functions of music, we interviewed 13 autistic adults and through a hermeneutic-phenomenological analysis found four overarching themes: Well-being, Identity and self-development, Connectedness, and Negative experiences. Findings show a broad and deep meaning of music in their lives, spanning from seemingly trivial functions such as making chores more enjoyable, to existential questions, such as choosing to stay alive. We discuss the often-overlooked negative effects or experiences of music, noting that positive and negative effects must always be addressed together if we are to use music to lower stress and support well-being.

“*It is interesting; there is not that much research which would turn directly to people with autism, but it feels like there is so much written* about *the people, not in any sort of collaboration with them! When can I read the results*?” exclaims Mira after our last interview. She is right. Even though the special place music can hold in a life of an autistic person was mentioned in the very first article about autism (Kanner, 1943), it was not until dozens of observational studies and half a century later that researchers turned to autistic people and *asked* how they perceive music and what it means to them (Allen et al., 2009; Bakan, 2018).

## Meanings, uses, and Functions of Music

People use music to satisfy a great breadth of needs and socio-cultural functions. They span from entertainment and aesthetic enjoyment to enforcing conformity to social norms, (DeNora, 2016; Hallam et al., 2016; Macdonald et al., 2012). In this article, we will focus on its meanings and functions from the individual’s perspective.

Schäfer and colleagues (2013) set out to create a comprehensive list of the psychological functions of music listening. Through reviewing existing literature, they found 129 distinct functions, which they distilled into three main categories: Arousal and mood regulation, Self-awareness and Social relatedness. There are several other such categorisations (e.g. Greb et al., 2018; Groarke & Hogan, 2016; Maloney, 2017), but most of them agree that people listen to music to support their well-being and tackle different daily demands, to feel aesthetic enjoyment and connectedness, and to have a space for reflection and self-development.

In a recent meta-ethnographic study, Perkins and colleagues (2020) looked into how participatory music engagement (e.g. playing in a band, singing in a choir) supports mental well-being. They found that making music helps manage and express emotions, facilitates self-development, offers respite and promotes connections (with people, heritage, and past).

While rarely mentioned, not all effects of music are positive. Apart from the more obvious physical harms, such as loss of hearing, there seem to be many additional, less readily observable ways of harming. Silverman and colleagues (2020) developed a theoretically informed model of music-induced harm, drawing attention to seven areas of potential harm: physical (e.g. musicogenic epilepsy or seizures), affective (e.g. mood worsening, anxiety), behavioural (e.g. maladaptive coping strategies, such as denial or disengagement), cognitive (e.g. impaired focus of attention), identity (e.g. intimidation), interpersonal (e.g. isolation) and spiritual harm (impaired ability to experience meaning in life). But so far both positive and negative effects and uses of music have rarely been explored in marginalised groups, such as the autistic population (Bakan, 2018).

While it is still not fully understood *how* music affects us in such profound and diverse ways, we are getting more insight into underlying neurophysiological mechanisms. Music for example affects neurotransmission in the brain´s reward pathways, as well as hormonal levels via hypothalamic-pituitary axis (e.g. lowering blood levels of cortisol). It has also been shown to affect blood pressure and heart rate, increase immunological measures, stimulate neural coherence, and impact hormones related to social affiliation (for overview see Chanda & Levitin 2013; Clements-Cortes & Bartel, 2018; de Witte et al., 2020; Legge, 2015).

### Music and Autism

In the first article about autism, Kanner (1943) noted that many of the autistic boys he observed seemed to have been interested in music and had strong musical abilities. In the decades following these early observations, researchers dug deeper into the (often superior) musical abilities of autistic people as compared to non-autistic, and into their physiological and neurological responses to music (for an overview see Quintin 2019). Their subjective experiences of music have remained largely unexplored (Bakan, 2018).

Another section of research on music and autism is focused on the effects of music therapy, which has been used for this population since the 1940’s (Reschke-Hernández, 2011). In a recent systematic review, Marquez-Garcia and colleagues (2022) conclude that, because of methodological weaknesses of the past studies, they cannot say for sure whether music therapy has any effects on the psychosocial functioning of autistic people. What is arguably more pressing than the methodological weaknesses, are the outcomes that a lot of the studies are focusing on. They measure the efficacy of music therapy with diagnostic tools such as ADOS or ADI-R, and other observational tools focusing on autistic “symptoms”, such as the frequency of eye-contact (Marquez-Garcia et al., 2022). Such goals are not at all aligned with the needs and wishes of the autistic community.

“*Changing behaviour, as such, should not be the main goal of clinical research or treatment for autistic people of any age. Appearing autistic or acting in typically autistic ways should not be considered an illness,”* (Pukki et al., 2022, p. 5).

There is a pressing need to explore autistic people’s subjective experiences of music, integrate those with the results of observational studies and include the autistic population in the discourse on what are important outcomes of music-based interventions.

### Subjective Experiences of Music in Autistic Adults

The first (and so far only) study of subjective experiences of music in autistic adults was done by Allen et al., (2009). The authors found that autistic adults most often use music to alter or match their moods and as a therapeutic tool (e.g., to ease emotional pain). In addition to that, music made them experience belonging, aesthetic pleasure and enjoyment, as well as a sense of achievement in the cases where participants were musicians. Interestingly, the participants used music to change the arousal of their states (e.g. from calm to excited or vice versa) but not the valence of their states (e.g. from a negative mood or emotion such as sadness to a positive one such as happiness or vice versa) and had difficulty verbalising emotional responses to music. The authors explained their results by pointing out that, in comparison with non-autistic people, the autistic population has higher levels of alexithymia, which are difficulties in identifying, and describing feelings, as well as externally oriented thinking (Preece et al., 2020; Watters et al., 2016). It is important to note that all participants in Allen’s study (2009) were able to express themselves in writing and the characteristic of their autism were not known. The questions were focused on positive aspects of music (e.g. “what about music makes you want to listen to it?”, “Does the music ever help you understand what is going on when you watch films or TV?”) and never explicitly asked about negative aspects of music.

The immense heterogeneity of the subjective experience of music can be seen in greater detail in the book by ethnomusicologist Bakan (2018) and his co-authors, autistic individuals for whom music plays a central role in their lives. Through conversations (which in some cases spanned several years) the book follows their life narratives and touches upon many ways of experiencing the world through music, from learning how to interact with others as a child to expressing oneself as a professional musician.

Matsuno and colleagues (2020) looked more specifically at how autistic adults (fluently conversing, with no intellectual disability) engage with music through music-listening technology. Informants used online platforms (such as Spotify or Youtube) to search for music, get recommendations, make playlists, listen to albums and follow different artists. Besides playing an instrument and making their own music, participants listened to music while commuting, working, gaming, sleeping, waking up, thinking creatively and coping with stress and negative emotion. However, they have not explicitly asked about possible negative aspects of music, and only focused on the positive (e.g., *“*What kinds of music do you like, and what about it do you enjoy?”). We see that music can be a large part of a regular day in the life of an autistic individual, which is why knowing more about their experience of it, can make society more understanding and inclusive when creating spaces for musical expression and engagement (Bakan, 2018).

The latest addition to this area is a study by Kirby & Burland (2021). They conducted interviews with 11 autistic adolescents and young adults (aged 12 to 25 years). The participants had secondary diagnoses such as learning disabilities, mood disorders, or Down syndrome, and were allowed to communicate with the interviewer in Picture Exchange Communication System (PECS) typed/written words and questions, Makaton signing or verbal communication. Authors found that the participants use music for four broad functions: cognitive (e.g., for support of other activities or a distraction), emotional (e.g., to manage their emotion, express them and reminisce), social (e.g., to develop relationships, and social skills) and those connected to identity (e.g., formation and communication of their identity to others). In contrast to the study by Allen and colleagues (2009), participants in this study did not only use music to change the arousal of their states but also the valence (e.g. from sad to happy). They concluded that autistic adolescents use music in similar ways and for similar reasons as non-autistic populations. Authors did not report any negative aspects of music, but as they do not provide the questions used, we do not know whether that was because of the focus of the questions, or because the theme simply did not appear.

The evidence we have so far on subjective experiences of music in autistic adults is scarce, and many areas remain to be explored. We know very little e.g., of how they experience music engagement in group settings, or in which contexts music could be a source of stress for them. Furthermore, there is very little information from autistic communities outside of the UK and USA.

### Aim of the Study

This study aims to explore the different meanings and functions of music in the lives of autistic adults. The autistic community also needs and desires more research on stressful environments, which is why special attention is paid to the negative effects of music. These insights could help practitioners develop and adapt existing music-based interventions, as well as more broadly, give a perspective on how we can integrate inclusive creative spaces into our society and inform recommendations regarding e.g., access to headphones or music platforms, music in school/workplace, music in public places etc. Our research questions are:

RQ1: What are the roles of music in the lives of autistic adults?

RQ2: Under which circumstances can music be a source of stress for them?

## Methods

We obtained ethical approval from the Swedish Ethics Authority (Etikprövningsmyndigheten), under the application code 2021 − 01121. We informed the participants about their rights, the procedure, and the aim of the study, and they freely gave their written informed consent.

### Recruitment and Participants

We invited autistic adults to participate through a short project presentation in a Swedish magazine (Riksförbundet Attention) focused on neuropsychiatric conditions, as well as an invitation on the webpage of the Karolinska Institute. To be included in the study, participants had to be 18 years of age or older, have a formal diagnosis of autism or Asperger’s syndrome, had to be able to provide their own legal informed consent, as well as be fluent in spoken Swedish. It did not matter whether they were – or how they were engaged with music to be included. They were aware, that the study will be focusing on music and well-being.

Thirteen autistic adults participated in the study (seven identified as female and six as male). Their ages ranged from 24 to 69 (mean age was 37, SD 12.49). Seven of them were employed, three of them were students, one was unemployed, one in organised daily activities and one pensioner. At the start of the interview, participants were asked which formal diagnoses they have received. We did not have access to their medical records to check their answers. They were engaged with music in different ways; one of them made music professionally, three of them composed and recorded their own music as a hobby, five of them had hobbies connected to playing, singing, or dancing, and four of them only engaged with music through listening. Their data was saved under unique codes, and for the writing of the article, we gave them pseudonyms. More information about the participants’ characteristics can be found in the Appendix. We scheduled interview meetings with the first seven women and seven men that applied for the study (14 in total, but one of the men did not get back after the first interview). This number was based on our available resources, and we put the rest on a waiting list, in case we would see that the data is not saturated and very diverse. After looking at the interviews of the 13 participants we decided, that the data seemed saturated enough.

### Interviews and Procedure

We conducted two semi-structured interviews with each participant via the video-conferencing platform Zoom. The first interview was on average 26 min long (SD = 11.37) and the second 28 min (SD = 9.96). No one besides the participant and the interviewer was present. The reasons for the two interviews were the well-being of the participants (we did not want to make the interview too long and tiring for them) and the depth of information (we wanted to prepare new questions stemming from their answers and allow them to reflect on the topic and share insights they might have forgotten to share in the first interview). The interviewer, who has a PhD in special pedagogy in music, was not involved in the planning of the study or the analysis of the interviews. She did not meet the participants prior to the interviews. She was experienced in interviewing and working with people with disabilities, and used an adaptive style of interviewing, where she followed a list of questions, but made adaptations following the needs and answers of the participants. The list of interview questions is in the Appendix.

The audio and video of the online conversation were recorded with the participant’s consent. We auto-transcribed the interviews using the platform Amberscript (Amberscript Global, 2022) and then checked and corrected the transcripts manually. We used NVivo (QSR International Pty Ltd., 2020) to code and analyse the transcriptions.

### Data Analysis

We used a hermeneutic-phenomenological approach (Dibley et al., 2020; Ricœur & Thompson, 2016; Tan et al., 2009) to analyse and interpret the interviews because it can unveil the meaning of experiences and is well suited for presenting situated narratives, which in this case have not yet been thoroughly heard. These types of approaches have been found to work well for autistic populations (Howard et al., 2019; MacLeod, 2019; Nilsson et al., 2019).

We aimed to reveal an interpretation of the meaning that music engagement has for autistic adults. The researcher who conducted the analyses wrote a reflection about her pre-understandings and by acknowledging them, strived to adopt an open mind and take her pre-existing knowledge into account (Crowther et al., 2017; Dibley et al., 2020). She has a master’s degree in psychology and a background in Western classical music. She has previous research experience with autistic children in the context of music therapy. At the time of the analysis, she was a doctoral student in the field of music and health. She identifies as non-autistic. According to Ricœur’s view of hermeneutics, the researcher’s existing knowledge and the data from the text are going to be unavoidably intertwined in the interpretation (Ricœur & Thompson, 2016; Tan et al., 2009). While the analyses were mainly conducted by the first author, the third author joined at the end of each step and iteration. All three authors discussed the final selection of themes. Participants were not asked to check the themes and findings as themes and interpretations in hermeneutic phenomenological stem from a whole-picture perspective which they did not have access to.

We started the analysis with *naïve reading*, which meant reading each transcript, summarising it and noting our initial impressions and preliminary themes. We then prepared the text by marking *meaning units* (smallest parts of the text that answer the research questions) and summarising them into *condensed meaning units*. We looked for connections between the condensed meaning units, combining them into *themes* and *sub-themes*. We kept re-reading the transcripts until we reviewed and verified all themes. Finally, we integrated the findings into a complete interpretation (Dibley et al., 2020; Ricœur & Thompson, 2016; Tan et al., 2009). In striving for rigour and trustworthiness, we made the process of data collection and analysis as transparent as possible, engaged in reflexive self-awareness, and included verbatim quotes to present the richness and robustness of the data (Lincoln & Guba, 1982).

## Results

As described in the methods section, we started the analyses with *naïve reading.* This served as the basis to which we kept returning throughout the process, to be aware of how our first impressions might be shaping our understanding of the transcripts. This is the summary of initial observations:

- Music is a valuable coping aid.
- Participants use music to cope with and manage emotion (to induce it, change it, strengthen it, distract from it etc.)
- Participants use music as an aid to their executive functions (directing, maintaining attention, starting a task etc.)
- Life without music is seen as dull, empty, cold, and boring.
- Participants use music to affect their nervous system arousal.
- Music can be a source of stress in certain situations.
- Music can give a sense of control, and agency.
- Music can be a way to connect with others, as well as with oneself.

When searching for meanings of music engagement, we found four themes: *Well-being and coping*, *Identity and self-development*, *Belonging and connecting* and *Negative experiences*. These themes and their sub-themes are presented in Table 1.

**Table 1:** Overview of the found themes and subthemes

*Themes*	*Sub-themes*
Well-being	1) Emotions and physiological arousal 2) Cognitive functions 3) Auditory environment 4) Respite 5) Hope and perseverance
Identity and self-development	1) Identity and self-expression 2) Creativity 3) Curiosity 4) Self-competence
Connecting	1) Connecting with others 2) Sharing experiences 3) Sense of belonging
Negative experiences	1) Emotion 2) Characteristics of the music 3) Setting 4) Critique

### Theme I: Well-being

First and foremost, music seems to be a tool for our interviewees to cope with the demands and challenges of their environments, and to take care of their well-being. There are five subthemes which illustrate the support they get from music: *Sound environment, Emotions and physiological arousal, Cognitive functions, Respite*, and *Hope and perseverance*.

#### Sound Environment

Music gives our interviewees agency over their sound environment, allowing them some control over the stimuli they are exposed to, and the possibility to block unwanted sounds. “*Yes, [I feel] better*”, says Britt, “*because I can like … I believe it has to do with impressions, that I can steer what it is that I am perceiving by listening to music*”. And this support can be especially important in more tasking environments; “*If I forget to bring my headphones when I am commuting, it is of course going to be a tough journey*” says Dan. But for some of them, music is also a way to connect with the sounds that surround them. Carl explains how he became fascinated with everyday sounds such as that of an escalator, a busy sidewalk or a metro arriving at a station when he was young. Although some sounds can be very exhausting and draining for him (“*I both hate and love sounds*” he says) he finds those that intrigue him. He often samples different sounds he encounters and combines them into soundscapes. As he puts it: “*It becomes a diary. An emotion-diary*”.

#### Emotions and Physiological Arousal

All of our interviewees, in one way or another, use music to affect their physiological arousal and emotion. Music helps them calm down when they feel excited, or get energised when they feel lethargic. “*It can energise my whole body or it can put me practically to sleep*” describes Frida. They are well aware of which types of music work for what need, explaining the importance of tempo, rhythm, loudness, and contrasts. When they cannot change the level of their excitement, they can sometimes change the valence, from negative excitement, to positive. Arne explains it like this: “*Even if the music does not calm me, it makes the experience more pleasant. […] It can change my agitated state […] to something more neutral or even positive. Like ‘Okej, I am still worked-up, but in a positive way’* ”.

They also use music for more complex changes in their states, such as to elicit new emotions (mostly positive emotion or pleasure; “*An emotional sushi. A fountain on the inside!* [laughter]” - Frida), as well as to gain some control or manage emotions they are feeling in the current moment. Helena says: “*I remember an experience when I had a panic attack and I got to dance and it just became manageable”.* Furthermore, music helps them process and express emotion. “*It [music] can be helpful because one can express emotion in a different way*,” says Mira. Emil describes the role of music with the following metaphor: “*I experience a sea of emotion. I have to be able to choose which are constructive in the moment. And music helps me – it is like a vessel, like a boat*”.

However, music does not only offer them agency over what they are feeling and how they express it – when this control fails it validates their emotion, as they “*recognise oneself in the different parts*”, as Iris puts it. Jonas describes it thus: “*It is like home ground or something … many people who have darkness inside them, can feel some sort of consolation in darker things [relating to art that expresses sadness, anger etc.]”*.

#### Cognitive Functions

In addition to emotion, our interviewees use music to support their cognitive functioning. They mainly use it to direct and maintain their attention on a specific task. “*I concentrate better*,” says Britt while noting also that lyrics can disrupt her focus with tasks like writing. Lovis says music helps her concentrate while she studies, but some participants also use it to inform their routines. “[*Music] helps me structure my day*”, states Dan and Kim explains: “*I have a playlist with three songs; I listen to these three songs and afterwards I have to start with whatever it is that I must do*”. Additionally, listening to music *while* doing chores (or other tasks they perceive as unpleasant) makes the chore more enjoyable and thus more likely to get done. “*It [music] can even energise me to be able to do stuff which I otherwise maybe would not do, like clean the apartment and such*,” admits Britt.

#### Respite

But music doesn’t only help in *doing* things, it can also help in simply *being.* More specifically, it helps our interviewees to be in a safe space and get some rest from their everyday lives. What Frida appreciates is that when she listens to music “*it is just me and the music in away*” and nothing else. It offers respite from the demands of the world (“*It’s a way for me to […] disappear into the music*” - Mira) or inner turmoil (“*When I feel bad, I can use it as a distraction*” - Lovis). This is not necceserally a sustainable way to address negative emotions or thoughts but might offer some temporary relief. Frida reflects: “[*One can use] music to just escape instead of sitting down and just being with oneself. Now that I am more comfortable in stillness, just being with myself, music doesn’t feel that necessary, but it is still very pleasant*”. Music can also offer a safe space by providing predictability and familiarity. Kim claims: “*it is the fact that I can put a song on repeat that gives me the feeling of calmness. It is not like a certain genre that would calm me*.” Mira explains this further, saying “*When I commute […] it is so nice to have a playlist with songs I know. It gives a sense of predictability […] and this is calming*.” For Helena, dance has a similar steady calming presence, which she greatly appreciates: “*It (dancing) becomes this safe routine that I can do no matter how I feel. I don’t have to have fun every time, or it doesn’t have to be fantastic, it is just that I do it because it feels safe and familiar, and I can do it even when I am having a bad day*.”

#### Hope and Perseverance

As you can see, the applicability of music for well-being can be quite broad. It can be used as an aid for seemingly trivial things such as studying and doing laundry, but with the variety of strengths that the interviewees can draw from music and the profoundly pleasurable experiences, it can take on a broader significance in one’s life. It seems to be a promise one can look forward to. “*In the future, I am looking forward to learning – to digging deeper into, for example, country, which I have not listened to at all […] and I am just looking forward to it all; to know that I will get to experience all this in the future*,” says Helena with a lively smile on her face. But this promise of joy can stay strong even in the worst of moments. Jonas revealed how essential music is for him: “*I used this strategy several times. I think ‘But I don’t want to miss this movie or that album’ […] I will not get to experience it if I throw in the towel now. […] so, in that way music and culture have helped me to … yeah*.”

To sum up, the interviewees use music for coping and well-being by gaining more control over their sound environment, by having an additional channel to regulate, process and express their emotions, as well as to regulate some of their cognitive functions, and lastly, to have a safe space to retreat to.

### Theme II: Identity and self-development

While music can be a versatile tool, it is more than just that. It seems to be closely connected to our interviewees’ identity and self-development, where we have found four sub-themes encompassing *Identity and self-expression*, *Creativity*, *Curiosity*, and *Self-competence*.

#### Identity and self-expression

Our interviewees see music as “*an extension of oneself*” (Dan), or for example, as having the music “*inside*” themselves (Carl). Jonas elaborates: “*My music - what I create - is me, but in the form of music […] There is so much to find. If one breaks it down and looks at the pure structure and its parts, there is so much to find that is me*”. They also use it to express themselves, in Carl’s case with “*sound diaries*”, in Dan’s, Gustav’s and Jonas’s with compositions, in Mira’s with the choir singing, and in Kim’s case, it takes the form of dance.

#### Curiosity

“*And sometimes I listen to music simply because it is interesting*!” says Dan, smiling. Each of our interviewees seems to be drawn to something else; some to the intricate details such as those of baselines or harmonies, others to the music piece as a whole, some to the story behind the lyrics, while others don’t notice the lyrics at all (Frida). Although, as we saw earlier, predictability can be an important and reassuring part of the musical experience, music can also be a comfortable way to embrace novelty. Emil compares music to “*keys*” that unlock different experiences. “*People talk about Asperger’s and autism – how I should like sameness, but I am constantly on the look for new music”* reflects Frida.

#### Creativity

Some of the participants composed, made digital music, played instruments, sang or danced and being able to express themselves creatively was very important to them. “*It is probably precisely in this [creative] process that one gets to be a bit free, like … to have one’s own space*” explains Mira. When asked what would it be like without music, Gustav said it would be “dull”, but that it would be necessary to have another outlet for his creativity.

However, they did not always use music as a channel for their creative expression. Sometimes they used it as an inspiration to do other (non-musical) creative tasks. Britt, for example, uses it for her writing: “*It can be motivating and inspiring [listening to music] and can prevent you from getting stuck in a text*”. And Dan who paints says: “*I use music to get my creative process going*”.

#### Self-competence

The outcomes of these creative processes can also give a sense of self-competence and self-worth. Gustav shares: “*There aren’t many other things I did that I actually finished. [With music] I get to complete something when nothing else gets done*.” It can be a source of pride and Jonas, now working on his fifth album says: “*You get this amazing feeling when you made something yourself and think ‘Oh this sounds good*!’”. One could argue whether they have created their works and developed their music skills *despite* or *because* of the challenges they face. Jonas believes the latter: “*I think I might have my diagnosis to thank for my musicality and creativity*”. This theme was not only present in those who perform or make original music, but also in those who play, sing or dance, for their own pleasure.

To summarise, our interviewees see music as a part of their identities, and as a skill, they can be proud of. They use it to express themselves, spur their creativity and feed their curiosity.

### Theme III: Connectedness

#### Connecting with Others

Music seems to help our interviewees to connect with others, especially when they don’t know others that well. Music, or an art activity, can make it easier to know what to talk about. For Dan, exhibitions and concerts are very important: “*It might be my only real chance to connect with someone else. […] It is something very concrete and something I can talk to them about.*” Similarly to Kim who says she “*would not nearly know as many people*”, were it not for music, Frida enjoys the opportunities to connect during her choir practice: “*We are all working toward the same goal. […]And then it doesn’t really matter that I [otherwise] do not know how or what to talk about”.* For others, music makes it easier to connect without relying on verbal communication. Helene explains why she likes dancing: “*I am quite a social person, and also autistic. […] it can be so relaxing to just go out and dance, hanging out with others, have music surrounding you, and avoid having to impress others with a lot of words and interesting discussions*”.

#### Sharing Experiences

Music is also a way to share pleasant experiences with friends and loved ones. Dan sometimes starts to “*spontaneously harmonize*” with his partner, looking for strange intervals, grinningly saying it is “*their thing*”. Lovis and Mira like to hang out with their friends while listening to music, and Gustav has started exchanging music ideas with a few of his coworkers.

Music also has a place with more negative experiences. Jonas tells us how important a specific band was for him. Their lyrics have themes of marginalisation and mobbing, and he says their music “*meant a lot*”. “*When you feel bad, it is nice to hear someone else [feeling similar]. It is like you connect somehow, but through music”* adds Frida.

#### Sense of Belonging or Connections

There also seems to be a broader sense of belonging and connection, not to specific people, but to humanity, nature or to the music itself. To Emil, music is a “*key*” to nature, noting the parallels between musical works and bird songs. Dan explains how he relates to music: “*You get some sort of connection, resonance with what you listen to*”. In Mira’s case, it can extend to humankind: “*With certain songs, I just feel connected to everybody, I don’t know how to explain it*”. And for Helena, dancing is a way in which she can “*pull off relating to large crowds of people in an enjoyable way*”.

When asked whether they would enjoy organised group-based music activities with other autistic people, their answers were different. Dan admitted it would likely be “*too intense*” for him and Frida liked the idea, saying it is easier for her to connect with other autistic individuals. As one can expect, there was no unanimous answer; some people disliked the idea, others thought it would be great, but they mostly agreed that the groups would have to be small.

In short, music seems to aid our interviewees in connecting to other people, known or unknown, sharing experiences, as well as to feel a sense of belonging or connection.

### Theme IV: Negative Experiences Connected to Music

Music can also be connected to negative experiences, which were largely overlooked in past research. According to our results, they can either stem from the music itself (like its characteristics or the emotions it might spur) or from the context of music engagement (such as an uncomfortable setting or the fear of being evaluated by others).

#### Characteristics of the Music

”*Some music you just cannot listen to*”, says Emil, which seems to be a common sentiment among our interviewees. They are mostly bothered by three things: the “wrong” genre, the volume of the music, and the poor quality of sounds (like crackling in the speakers) or of the music itself (like wrong tones). “*Others – they seem to tune these things out*,” reflects Frida, who explains how frustrating humming noises in loudspeakers can be. “*It is just me, and a coworker who is educated as a sound technician, who seems to notice it*”. This is not a problem as long as one gets to choose *how* and *what* music to listen to, therefore, the lack of agency seems a crucial ingredient in most negative experiences with music.

While we can all feel uncomfortable with music sometimes, it is important to keep in mind that some people are especially sensitive to stimuli and have a hard time filtering them out. “*I do my best to avoid such situations [where he does not have control]*,”, shares Arne, and Gustav adds “*I feel the need to leave, even if I am at work. But I have learned to stop myself*”. It is difficult to imagine what another person feels, but Frida paints quite a descriptive picture: “*It is difficult to explain […] It is like a-room-full-of-snakes uncomfortable”.*

#### Emotion

The emotions music can incite in an individual, can *also* be perceived as an unwanted, negative experience. For Frida and Iris, music in movies can be sometimes too emotionally intense. “*I get this physical feeling, often in my stomach […] If I knew this would happen in advance, I would not watch that movie,”* says Iris. Dan remembers intense experiences from concerts, one of them of Tchaikovsky’s Symphony Pathétique: “*There were a few times when it was almost too difficult to stay at a concert because I was so moved by music*”. Emil explains: “*It [experiencing art] is very draining. You take in so much at the same time […]”.* But it seems it is not only the fact that one cannot simply change the music in a movie theatre, a concert or at a party; even when listening to music at home, it can become too emotionally intense. Lovis, Frida and Iris describe how sometimes, one can choose the “wrong kind” of music for how one feels at the moment or how it can *become* too intense as one’s needs change. “*Then I think ‘Now I should really listen to something else so that I don’t intensify the emotion unnecessarily*”.

As strange as it may sound, music can also be *too enjoyable*. Dan and Jonas tell us they often fall into hyperfocus when composing and neglect their daily routines and other tasks. “*I fall out of my routines – be it eating or exercise – and they are important for everyone, let alone people like me*”. Then there is also another aspect. Frida tells us she feels so euphoric sometimes, that she experiences a “*crash*” when she is done listening to music, and she has to “*come back down to Earth*”. Experiences like these seem to be tied to the fact, that music is so strongly enjoyable and rewarding, and in our view represent the other, negative side of that same coin.

#### Setting

The negative experiences can also stem from the context of engaging with the music, and not the music itself. For one, listening to music on noise-cancelling headphones can be dangerous, as Jonas and Frida point out, as one doesn’t hear what is going on around them. But being highly aware of other things that are going on, can *also* be stressful. As Kim puts it, it is often not the music or dance which are stressful, “*it’s all the things that are happening* around *dance and music*”. Like not knowing how many people will come to a concert or what the venue will look like, trying to follow unclear instructions in workshops, trying to figure out the norms of social dancing, or simply having to be in a crowd of people. And while our interviewees might enjoy a particular song otherwise, sometimes the setting is simply wrong; like when one tries to focus on a conversation or on shopping in a store. “*It is very difficult for me to shut out stimuli*,” says Arne, adding that he tries his best to avoid stores with music, like most of our other interviewees. Mira explains: “*I can totally get stuck in the music*”.

#### Self-doubts/Fear of Evaluation

“*Don’t laugh*”, says Carl nervously before showing the interviewer his latest soundscape. As much as he enjoys combining sounds into music, he never shares it with others. For Mira, there is always the question of “*how are the others going to perceive it [my art]?”.* While some of the interviewees got a lot of support in their musical path, not everyone was so lucky. As a girl, Frida was told to sing quietly by her choir teacher, and afterwards always felt scared and nervous while singing. She explains: “*When you have neuropsychiatric conditions like ADHD or autism, you develop a little differently [certain areas faster than others], and in music skills, I just happened to be behind. […] When they spoke about measures, for example, I didn’t get anything! But then one day, it just clicked”.* For our interviewees, in the package of making music, there seems to also come the questioning of one’s musical skills, feelings of inadequacy, or anxiety about being judged by others. While Jonas believes making music is therapeutic for him, he also sees how stressed and anxious it can make him. “*I can be quite self-critical, and the more albums I release the worse it gets, it would seem*”.

To sum it all up, agency is an important part of whether a music experience is going to be perceived as stressful or not. A disliked genre, high volume, or low quality of sound can be very frustrating, especially for people who have a harder time filtering out stimuli. Music can also evoke strong, negative emotions, be it from the listening experience or by expecting criticism from others. The unfamiliarity, complexity, or intensity of a situation surrounding music, can also be a large contributor to stress.

### Complete Interpretation: Two Sides of a coin

For autistic adults, music can be an important tool in overcoming a diverse palette of challenges. From directing attention and regulating emotions to spurring one’s creativity or having a safe space to take a break from it all, music seems like a rich resource for our participants. Furthermore, it extends beyond its immediate functions and gives additional meaning to the person and their life. It is an important channel for marginalised people to express themselves authentically as well as a bridge to other people, humanity and nature.

But precisely *because* it has such a strong impact on a person, and is so close to our emotions and to who we are, music also has considerable potential for harm. We named the complete interpretation arising from the interviews *Two sides of a coin*. With this, we would like to draw attention to the fact that the use of music is not beneficial per se, but is highly dependent on the context. For example, while our interviewees can elicit pleasant emotions with music, they can also unexpectedly elicit negative emotions, or while music can help them process emotion, they can also get stuck in rumination. Similarly, music can provide them with a comfortable space of respite, but it can also make them disconnect from their environment as well as their negative thoughts or emotions. And while it helps them connect and communicate with other people, it also makes them vulnerable to other people’s judgement and easier to isolate. We have to be equally mindful of the positive and negative aspects of music engagement.

## Discussion

Using in-depth interviews, we looked into how autistic adults perceive the roles of music in their lives. We found four overarching themes: *Well-being*, *Identity and self-development*, *Connection* and lastly - a theme which is largely overlooked in the existing literature - *Negative experiences*.

Our findings align in several areas with those of Allen and colleagues (2009), and with those of Kirby & Burland (2021) even though the latter involved autistic adolescents and not adults. In both of these studies, as well as in ours, the participants used music to support their well-being by managing their arousal and employing music for its “therapeutic” properties (as called by the participants), they used it to reflect upon- and express their identity and they used it to get a sense of connectedness with others.

However, when it comes to emotion management with music, our findings differ. Allen and colleagues (2009) report that their participants almost exclusively used music to change the arousal of their states, but not their valence. In the study by Kirby & Burland (2021), as well as in our study, this was not the case. Our participants talked about managing, processing and eliciting a wide range of emotions with the help of music (e.g. anger, frustration, sorrow, euphoria). This discrepancy could be due to the fact that, while alexithymia is highly prevalent among autistic people, it is not present in all (Kinnaird et al., 2019). Since our sample sizes were small, Allen and colleagues (2009) might have had a sample with higher levels of alexithymia, explaining the absence of emotion-related vocabulary. Compared to the previous studies, our findings show a more nuanced understanding and range of functions, possibly due to methodological differences (we conducted two interviews with each participant, allowing us to reopen certain topics and further explore the phenomena).

Similar themes have been mentioned in studies on non-autistic populations (Greb et al., 2018; Groarke & Hogan, 2016; Maloney, 2017; Perkins et al., 2020; Schäfer et al., 2013), but we would like to emphasize several uses of music which we have not found in these studies. The first one was connecting with the sound environment through music. While many of our interviewees report being sensitive to sounds, one of them samples sounds he comes across in his daily life (sounds of elevators, metro etc.) and combines them into soundscapes, or “emotional diaries”, as he describes them. This seems to be not only a way for him to creatively express himself but a way to cope with and have some agency over the sound environments he is exposed to.

The second use of music, we would like to draw attention to, is the creation of predictability and familiarity. A wish for familiarity was briefly mentioned in the study by Allen and colleagues (2009), but they concluded that participants who listened to classical music had a wish for familiarity in music, while those who listened to pop did not. The interviewees in our study explained that music is not only calming or enjoyable because of its characteristics or memories connected to it. It is calming also (or in some cases predominantly) because of its familiarity and the feeling of predictability it provides. It does so in several different ways; the listener, can listen to a certain piece as many times as they want and in virtually any situation they want, but there are many more subtle aspects of predictability in music (e.g., repetitions within a piece, and common tonal and rhythmic frameworks across music styles). The need for predictability is highly prevalent in autistic people (American Psychiatric Association, 2013), and music seems to be an accessible tool to sate this need. On the other hand, music fed our participants’ curiosity and allowed them to be exposed to novelty in a safe environment they had control over.

Another use of music, which is not often mentioned in the literature on non-autistic people, is use of music as an aid in establishing routines. Similarly to Kirby & Burland (2021), we found that our interviewees use music to keep track of time, to know *when* to start, and to make it easier to start with a routine. In addition to sustaining routines, music helps them to connect with other people in a way that does not require verbal communication (e.g. through dancing together), or by providing them with a topic to discuss. This use was not mentioned in the previous literature and seems an important function for a population where difficulties in communicating and interacting are common (American Psychiatric Association, 2013). Music helped our participants to feel connected beyond interpersonal relationships, with humanity and nature. According to Neff (2003) “common humanity”, or perceiving your own experiences as a part of a larger human experience, is an important component of self-compassion.

As we see, there may be some small differences between non-autistic and autistic people’s uses of music, but differences in uses are only a part of the picture. While the ways they use music might be generally similar, the impact of music on their everyday life could be different. Let’s take for example using music to block other sounds, which seems to be a common use in both populations. From our interviews, we can see that listening to music on one’s daily commute can, for an autistic person, be the deciding factor between going through with their plans or getting so overwhelmed by everything that is happening on the bus or metro around them, that they have to get off and go home. On the other hand, background music in stores was by some participants perceived as intolerable. If we want to create more inclusive public spaces, music is definitely a component we must address. Bradshaw & Holbrook (2008) offer a critical perspective on how culture is often used and degraded as means of social control. As music is evidently important to people with sensory sensitivities, it is vital to take into account the context and consequences of music use (or inability to use music) in addition to the strategies, which are more commonly studied (e.g. Laiho 2004; Lundqvist & Korošec, 2021). We believe that the themes we found point us towards more appropriate outcomes in music-based interventions, as opposed to measuring “autism symptom” severity. Judging from our results, we should further explore outcomes connected to coping with stress, perceived self-competence, emotion regulation, feelings of connectedness and executive functioning.

Perhaps the most important contribution of this study is the insight into the negative experiences connected to music, which are rarely mentioned in the literature (Silverman et al., 2020). So far, they have mostly been addressed as sound-related sensitivities in autistic children (e.g. Dickie et al., 2009; Kirby et al., 2015) and they can also be seen in our interviews with adults. Sound sensitivity, found in many autistic children and adults, can be debilitating (see Williams, He, et al., 2021; Williams, Suzman, et al., 2021) and is an important factor to consider when creating inclusive environments or creating interventions for this population (for example avoiding music in grocery stores, or allowing the headphones at work or school, to help drown out distracting sounds). We found that our interviewees can experience a range of negative effects of music engagement, stemming from the characteristics of music (e.g. genre, loudness), an inappropriate setting, intense emotions as a reaction to music and the fear of being judged by others. In many cases, the negative experience stems from the lack of agency over *what* music they listen to, as well as *where/when* and *how* (e.g. music in a store). However, their origin is sometimes more complicated. For example, while music engagement provided our participants with a creative outlet, feelings of flow, achievement, and connection to other people, it often simultaneously presented a source of stress and worries about the quality of their music skills and the fear of being judged by others. Musgrave (2022) who recently addressed this discrepancy, calls for more research focusing on the well-being of musicians and for a more “sophisticated appreciation of the *uses* of music and its impacts on wellbeing” (p. 1). Another polarisation can be seen in the use of music as a space of respite and distraction. Although its distraction might offer a brief break to rest and recover, using this strategy to deal with problems isn’t a sustainable, healthy approach (Baltazar & Saarikallio, 2019; Saarikallio et al., 2015; Silverman, 2020), which was also acknowledged by some of our interviewees. There seems to be a negative side even to the pure enjoyment of music. As our interviewees explained it, “*coming back to earth*” after an amazing musical experience can feel quite depleting or depressing.

Despite the complexity of its effects, our interviewees see music as important and meaningful, and their lives without it as “duller and emptier”. This study offers an illustration of how the uses of music can be neither beneficial nor harmful in and of themselves but are highly complex and context-dependent.

### Limitations

This study did not include participants who do not communicate verbally or who communicate through non-traditional methods, which means a part of the autistic population was not represented. As the area is notably under-researched, we decided to start with a more basic approach, employing virtual interviews, to get some initial insight into this population’s experiences connected to music. This study offers a good starting point, from which we can refine the study design and techniques, to make them tailored to a broader range of needs, characteristics and ways in which people express themselves (writing, picture cards, sign language etc.). Similarly, the majority of our participants were employed or studying, while those who cannot do that or are less integrated into the Swedish society, were underrepresented in our study. As the autistic population is very diverse, future studies can look closer into the subpopulations, and employ specific methods of recruitment, which will help them include a broader range of participants.

Adapting recruitment and data collection methods in the coming studies could grant us access to a more diverse sample of participants, but it is possible, if not likely, that people who are not interested in music or do not see great value in musical experiences, will not choose to participate in a study of this kind. It is important to keep this part of the population in mind and find a way to include their views, as brief as they might be. This is a subgroup for whom music interventions or activities might have to be adapted, or might not even be suitable if they have no interest in engaging in music.

#### Conclusions and Future Research

This exploratory study offers insights into the functions of music for autistic adults, emphasizing the importance of contextual factors. The positive and negative effects of music seem to stem from the same mechanisms, being two sides of the same coin. In the future, these findings should be tested on a larger, more diverse sample of people with different support needs and ways of communicating. It would be informative to include a non-autistic comparison group. As the negative effects of music in music-based approaches are understudied, we believe it is important to further investigate harms connected to music, which could help us predict and prevent any harm before it happens. We need more research on the subjective experiences of art in the autistic population, which are important in empowering individuals and building an inclusive society. We hope the insights will help establish more informed therapeutical and pedagogical approaches for autistic people and help create autism-friendly environments.
